# Assessment of Toll-like receptor 2, 4 and 9 SNP genotypes in canine sino-nasal aspergillosis

**DOI:** 10.1186/s12917-014-0187-6

**Published:** 2014-08-16

**Authors:** Elise Mercier, Iain R Peters, Frédéric Farnir, Rachel Lavoué, Michael Day, Cécile Clercx, Dominique Peeters

**Affiliations:** 1 Department of Veterinary Clinical Sciences, Division of Companion Animal Internal Medicine, Faculty of Veterinary Medicine, University of Liège, Sart Tilman 4000, Liège, Belgium; 2 Molecular Testing, TDDS ltd., The Innovation Centre, University of Exeter, Exeter, UK; 3 Biostatistics, Bioinformatics and Animal Selection, Department of Animal Production, Faculty of Veterinary Medicine, University of Liège, Sart Tilman 4000, Liège, Belgium; 4 Department of Clinical Sciences, University of Toulouse, INP, National Veterinary School of Toulouse, Toulouse cedex 03 31076, France; 5 School of Veterinary Sciences, University of Bristol, Langford House, Langford BS40 5DU, Bristol, UK

**Keywords:** Sino-nasal aspergillosis, Dog, Toll-like receptor gene, Single nucleotide polymorphism

## Abstract

**Background:**

The exact aetiology of canine sino-nasal aspergillosis (SNA) is unknown. In man, dysfunction in innate immunity, particularly in the function of pattern recognition receptors, is implicated in the pathogenesis of inflammatory sino-nasal disease and in fungal diseases. Associations between single nucleotide polymorphisms (SNPs) in Toll-like receptors (TLRs) and these diseases have been identified. Similarly, in dogs SNPs in genes encoding TLRs may be important in the pathogenesis of SNA. The aims of the present study were (1) to identify the presence of non-synonymous SNPs in the coding regions of the *TLR2*, *4* and *9* genes in dogs suffering from SNA, and (2) to investigate the SNP genotypes in dogs with SNA compared with a control population.

**Results:**

Direct sequencing of nine dogs of various breeds with SNA revealed two non-synonymous SNPs in the coding region of *TLR2*, eight in *TLR4* and four in *TLR9*. These non-synonymous SNPs were further evaluated in a case-control study of affected Golden Retrievers, Labrador Retrievers, Rottweilers and Beaucerons. Genotyping was performed using a combination of allele-specific primers and hydrolysis probe assays in 31 dogs with SNA and 31controls. No significant difference in minor allele frequency was identified between these groups, for all studied SNPs, in any of the four breeds.

**Conclusions:**

These findings do not support a role for non-synonymous SNPs in the *TLR 2*, *4* and *9* coding regions in the pathogenesis of canine SNA, but do not exclude a role for innate immunity in the pathogenesis of the disease.

## Background

The sino-nasal mucosa is an important interface with the environment and represents a dynamic system for innate host defence [1]. In addition to providing a barrier to the entry of pathogens, epithelial cells lining the airway are able to recognise pathogen-associated molecular patterns (PAMPs) [2]. Toll-like receptors (TLRs) are situated on these cells and on dendritic cell populations in this location and appear to play crucial roles in the distinction between commensal flora and PAMPs. Single nucleotide polymorphisms (SNPs) within the *TLR* genes have been reported to impact the immune reaction to microbial antigens, and to be associated with the incidence and outcome of infections [3]. Collectively, the TLRs cover the recognition of a wide variety of pathogens (i.e. viruses, bacteria, parasites and fungi). TLR2, TLR4 and TLR9 are particularly associated with recognition of fungi (e.g. *Candida albicans* and *Aspergillus fumigatus*) [4]–[9], and SNPs in these *TLRs* have been associated with higher risk of fungal disease in man [10].

In dogs, sino-nasal aspergillosis (SNA) is a cause of chronic rhinitis, and the disease is most often caused by *A. fumigatus*[11]. SNA is characterized by the formation of superficial mucosal fungal plaques within the nasal cavity and/or frontal sinus, associated with a severe mucosal inflammatory response and a local destruction of nasal bone [12]. As the disease usually affects systemically healthy dogs, and *A. fumigatus* is a ubiquitous fungus, a local immune deficiency in affected dogs has been suggested [13].

The clinicopathological features of SNA are similar to those of human chronic erosive non-invasive fungal sinusitis [14]. Although TLRs have not been studied in this type of sinusitis in people, they have been better characterized in chronic rhinosinusitis (CRS) including fungal CRS, and some forms of aspergillosis. Pathogenesis of the CRS complex remains unclear, but association of this disease with *TLR* polymorphisms, particularly *TLR2* SNPs, has been described [15].

Association between *TLR* SNPs and incidence of various forms of human *Aspergillus* infection have been reported. A significant association between the incidence of invasive aspergillosis (IA) in recipients of transplanted allogeneic stem cells and the presence of some *TLR4* SNPs in donors has been reported [16],[17]. Hence, Carvalho *et al.*[10] have described an increased frequency of chronic pulmonary aspergillosis among *TLR4* SNP carriers. In the same study, a SNP in *TLR9* was shown to predispose to the allergic form of pulmonary aspergillosis.

Polymorphisms in *TLR genes* have not yet been described in dogs with SNA, but alteration in these receptors could hamper their function, therefore disrupting the recognition of fungi, eventually leading to fungal escape from immune surveillance. Recently, up-regulation of *TLR2*, *4* and *9* mRNA expression has been reported in the nasal mucosa of affected dogs [18]. Comparison of the canine and human *TLR* genetic sequences show a high degree of homology [19]; suggesting the polymorphisms in *TLR* genes found in people with aspergillosis and/or fungal CRS might also be observed in dogs.

A retrospective analysis of the medical records at the Veterinary Clinic of the University (CVU) of Liège (Belgium) over the last 5 years revealed four predominant canine breeds diagnosed with SNA: Golden Retriever, Labrador Retriever, Rottweiler and Beauceron Sheepdog. A candidate gene approach was used to test the hypothesis that SNPs in *TLR2, 4* and *9* genes were associated with canine SNA. The presence of non-synonymous SNPs in the coding regions of the *TLR2*, *4* and *9* genes was investigated by direct sequencing of PCR-amplified products from these regions, using DNA from nine dogs with SNA belonging to various breeds (including the four aforementioned breeds). Secondly, a combination of allele-specific primers and Taq-man probe-based PCR genotyping was applied to samples from affected and control dogs derived from these four breeds in order to assess the significance of these SNPs in canine SNA.

## Results

### Selection of single nucleotide polymorphisms

The initial part of the study focused on a mutational analysis of *TLR2*, *4* and *9* genes in nine dogs with SNA belonging to various breeds. This analysis revealed two non-synonymous SNPs in the coding region of *TLR2*: *C137A* (*rs22410121*) and *C1547T* (not reported in the canine genome database). Eight non-synonymous SNPs were identified in the *TLR4* exons: *T23C* (not reported in the canine genome database but described by Kathrani et al. [20]), *C107T* (not reported previously), *T500C* (*rs22145736*), *A600C* (*rs22189454*), *A688G* (*rs22189456*), *G1039A* (not reported in the canine genome database but described by Kathrani et al. [20]), *A1571T* (*rs22124023*) and *G1807A* (*rs22123995*). Four non-synonymous SNPs were identified in *TLR9* exon: *G1138A* (not reported previously), *A1372C* (not reported previously), *A2158G* (*rs22882109*) and *G2927A* (not reported previously).

All non-synonymous SNPs identified resulted in a change in the class of amino acid coded (Table 1).

**Table 1:** **Amino acid change coded by non-synonymous single nucleotide polymorphisms in the canine** ***TLR2*,** ***4*** **and** ***9*** **genes**

**Gene**	**SNP (accession number)**	**Amino acid wild type**	**Amino acid change associated with SNP**
***TLR2***	*C137A* (*rs22410121*)	Serine	Tyrosine
	*C1547T*	Serine	Leucine
***TLR4***	*T23C*	Valine	Alanine
	*C107T*	Threonine	Isoleucine
	*T500C* (*rs22145736*)	Leucine	Proline
	*A600C* (*rs22189454*)	Histidine	Glutamine
	*A688G* (*rs22189456*)	Lysine	Glutamic acid
	*G1039A*	Alanine	Threonine
	*A1571T* (*rs22124023*)	Glutamic acid	Valine
	*G1807A* (*rs22123995*)	Glutamic acid	Lysine
***TLR9***	*G1138A*	Glutamic acid	Lysine
	*A1372C*	Threonine	Proline
	*A2158G* (*rs22882109*)	Serine	Glycine
	*G2927A*	Arginine	Histidine

### SNP genotyping and association study

To assess the significance of the identified SNPs, the allelic frequencies in a group of dogs with SNA belonging to four breeds commonly affected by SNA (Golden Retriever, Labrador Retriever, Rottweiler and Beauceron Sheepdog) were compared with those found in control dogs of the same breeds (Table 2), and no significant difference was found. Two SNPs (*TLR2 C1547T* and *TLR4 T500C*) were found monomorphic between and within samples from the four selected breeds.

**Table 2:** Single nucleotide polymorphism allele association with sino-nasal aspergillosis dogs

**SNP**	**Minor allele**	**Minor allele frequency in the 31 SNA dogs**	**Minor allele frequency in the 31 CTL dogs**	**p value**
***TLR2 C137A***	*C*	0.42	0.48	0.47
***TLR2 C1547T***	*T*	0	0	NA
***TLR4 T23C***	*T*	0.40	0.40	1
***TLR4 C107T***	*T*	0.03	0	0.15
***TLR4 T500C***	*C*	0	0	NA
***TLR4 A600C***	*C*	0.48	0.43	0.59
***TLR4 A688G***	*A*	0.47	0.43	0.71
***TLR4 G1039A***	*A*	0.34	0.34	1
***TLR4 A1571T***	*A*	0.13	0.10	0.57
***TLR4 G1807A***	*G*	0.13	0.10	0.57
***TLR9 G1138A***	*A*	0.06	0.05	0.70
***TLR9 A1372C***	*A*	0.50	0.37	0.15
***TLR9 A2158G***	*G*	0.55	0.43	0.21
***TLR9 G2927A***	*A*	0.03	0	0.15

Statistical significance was set at P < 0.05.

The same analysis was conducted within the four breed groups separately. Again, the allelic differences, although variable across breeds, were not related to the disease phenotype (Tables 3, 4, 5 and 6). In Golden Retrievers, the *TLR4 C107T* SNP was not found, and the least common alleles for all breeds taken together for *TLR9 A1372C* and *TLR9 A2158G* were the most common in this breed. In Golden Retrievers, the association of the *C137A* SNP with the disease almost reached significance. In the Labrador Retriever dogs, the least common alleles for all breeds taken together for *TLR2 C137A*, *TLR4 A600C*, *TLR4 A688G*, *TLR4* and *G1039A* were most common. In Rottweiler dogs, *TLR4 C107T*, *TLR4 A1571T*, *TLR4 G1807A*, *TLR9 G1138A* and *TLR9 G2927A* SNPs were absent, and the least common allele for all breeds taken together for *TLR4 A600C* was the most common allele. In the Beauceron Sheepdogs, *TLR2 C137A*, *TLR9 G1138A*, *TLR9 A2158G* and *TLR9 G2927A* SNPs were absent, and the least common allele in all breeds taken together for *TLR9 A1372C* was the most common allele in this breed.

**Table 3:** Single nucleotide polymorphism allele association with sino-nasal aspergillosis Golden Retriever dogs

**SNP**	**Minor allele**	**Minor allele frequency in the SNA Golden Retriever dogs**	**Minor allele frequency in the CTL Golden Retriever dogs**	**p value**
***TLR2 C137A***	*C*	0.15	0.45	**0.08**
***TLR2 C1547T***	*T*	0	0	1
***TLR4 T23C***	*T*	0.35	0.40	1
***TLR4 C107T***	*T*	0	0	1
***TLR4 T500C***	*C*	0	0	1
***TLR4 A600C***	*C*	0.30	0.25	1
***TLR4 A688G***	*A*	0.30	0.25	0.10
***TLR4 G1039A***	*A*	0.05	0.05	1
***TLR4 A1571T***	*A*	0.25	0.20	1
***TLR4 G1807A***	*G*	0.25	0.20	1
***TLR9 G1138A***	*A*	0.05	0.10	1
***TLR9 A1372C***	*C*	0.10	0.25	0.41
***TLR9 A2158G***	*A*	0.20	0.25	1
***TLR9 G2927A***	*A*	0	0	1

Statistical significance was set at P < 0.05.

**Table 4:** Single nucleotide polymorphism allele association with sino-nasal aspergillosis Labrador Retriever dogs

**SNP**	**Minor allele**	**Minor allele frequency in the SNA Labrador Retriever dogs**	**Minor allele frequency in the CTL Labrador Retriever dogs**	**p value**
***TLR2 C137A***	*A*	0.50	0.37	0.72
***TLR2 C1547T***	*T*	0	0	1
***TLR4 T23C***	*T*	0.44	0.25	0.46
***TLR4 C107T***	*T*	0.06	0	1
***TLR4 T500C***	*C*	0	0	1
***TLR4 A600C***	*A*	0.37	0.25	0.70
***TLR4 A688G***	*G*	0.37	0.25	0.70
***TLR4 G1039A***	*G*	0.50	0.31	0.47
***TLR4 A1571T***	*A*	0.12	0.06	1
***TLR4 G1807A***	*G*	0.12	0.06	1
***TLR9 G1138A***	*A*	0.18	0.06	0.60
***TLR9 A1372C***	*A*	0.37	0.19	0.43
***TLR9 A2158G***	*G*	0.56	0.37	0.48
***TLR9 G2927A***	*A*	0.12	0	0.48

Statistical significance was set at P < 0.05.

**Table 5:** Single nucleotide polymorphism allele association with sino-nasal aspergillosis Rottweiler dogs

**SNP**	**Minor allele**	**Minor allele frequency in the SNA Rottweiler dogs**	**Minor allele frequency in the CTL Rottweiler dogs**	**p value**
***TLR2 C137A***	*C*	0.39	0.17	0.26
***TLR2 C1547T***	*T*	0	0	1
***TLR4 T23C***	*C*	0.44	0.44	1
***TLR4 C107T***	*T*	0	0	1
***TLR4 T500C***	*C*	0	0	1
***TLR4 A600C***	*A*	0.39	0.56	0.51
***TLR4 A688G***	*A*	0.56	0.45	0.74
***TLR4 G1039A***	*A*	0.56	0.45	0.74
***TLR4 A1571T***	*A*	0	0	1
***TLR4 G1807A***	*G*	0	0	1
***TLR9 G1138A***	*A*	0	0	1
***TLR9 A1372C***	*A*	0.06	0.06	1
***TLR9 A2158G***	*G*	0.06	0.06	1
***TLR9 G2927A***	*A*	0	0	1

Statistical significance was set at P < 0.05.

**Table 6:** Single nucleotide polymorphism allele association with sino-nasal aspergillosis Beauceron sheepdogs

**SNP**	**Minor allele**	**Minor allele frequency in the SNA Beauceron Sheepdogs**	**Minor allele frequency in the CTL Beauceron Sheepdogs**	**p value**
***TLR2 C137A***	*A*	0	0	1
***TLR2 C1547T***	*T*	0	0	1
***TLR4 T23C***	*T*	0.12	0.37	0.57
***TLR4 C107T***	*T*	0.12	0	1
***TLR4 T500C***	*C*	0	0	1
***TLR4 A600C***	*C*	0.37	0.25	1
***TLR4 A688G***	*A*	0.37	0.25	1
***TLR4 G1039A***	*A*	0.25	0.12	1
***TLR4 A1571T***	*A*	0.12	0.12	1
***TLR4 G1807A***	*G*	0.12	0.12	1
***TLR9 G1138A***	*A*	0	0	1
***TLR9 A1372C***	*C*	0.25	0.50	0.61
***TLR9 A2158G***	*A*	0	0.37	0.20
***TLR9 G2927A***	*A*	0	0	1

Statistical significance was set at P < 0.05.

## Discussion

The present study investigated the association between SNPs in *TLR2*, *4* and *9* genes, and the risk of developing SNA in dogs. In the first part of the study, sequencing of the coding regions of these genes in nine dogs with SNA (from various breeds) revealed two, eight and four non-synonymous SNPs in the coding region of *TLR2*, *TLR4*,and *TLR9* genes, respectively. In the second part of the study, no association was found between these SNPs and the presence of SNA.

Some of the SNPs identified in the first part of the study were not found in samples from the four breeds of dog genotyped in the second part, and minor allele frequencies varied extensively between breeds, with some uncommon alleles in one breed becoming the most common alleles in the other, and vice versa. This supports a wide genetic variations between canine breeds [21].

The only result close to significance was found for *TLR2* C137A SNP in the Golden Retrievers. Due to the unpredictable direction of allelic frequency shift between dogs with SNA and controls, PLINK software was used to compute two-tailed Fisher’s exact test *p*-values. Using a one tailed test would have led to half the actual *p*-value (0.04; data not shown), thereby reaching significance. Although this might be interpreted as a significant result, the rationale for choosing a one-tailed test might be debated. Furthermore, multiple testing issues (i.e. performing several tests) could be considered, leading to a lowered significance threshold that would make this result not significant. On the other hand, the power of the test might be low due to the relatively small size of the sample. Therefore, this SNP could merit further investigation with a larger number of dogs.

Although some researchers have studied the association between TLR SNPs in human CRS and some fungal diseases, the results are heterogeneous and sometimes contradictory. Several researchers have shown a significant association between *TLR4* and/or *TLR9* SNPs and the incidence of several forms of aspergillosis [10],[16],[17]; however, another study failed to associate these polymorphisms with that disease. Recently, Carvalho *et al.* noted a positive association between *TLR4* SNPs and fungal colonization, but not susceptibility to fungal infection [22]. Kesh *et al.* did not report any positive association of recipient or donor *TLR4* SNPs with the incidence of IA in patients undergoing allogeneic stem cell transplantation [23]. In human CRS, there are also discrepancies between studies regarding the role of SNPs in *TLR* genes. Park *et al.* reported an association between *TLR2* SNPs in a Korean population [15], but Tewfik *et al.* could not confirm these results in Quebec [24].

The effect of the environment on susceptibility to certain diseases is another important aspect that should be considered together with genetic background. Vercelli [25] reported that genes and environment are intertwined in complex nonlinear relationships. Therefore, the same genetic background may result in the expression of different phenotypes when exposed to different environments. Consequently, in a disease with a complex pathogenesis, the genotype of multiple contributing genes, together with environmental factors, may modify the likelihood of an individual developing the disease. In such instances, individual gene mutations are insufficient to cause the disease on their own [26].

There are several limitations to the present study. Firstly, although we cannot confirm an association between polymorphism in the *TLR2*, *4* and *9* genes with SNA, it is possible that because of the small size of the study population, a slight/weak but real contribution of *TLR2*, *4* or *9* SNPs has been missed. Another potential limitation is the selection of control dogs. Although efforts were made to exclude dogs with respiratory, inflammatory, immune-mediated, neoplastic or infectious diseases, the possibility cannot be excluded that these dogs might develop such disease later in life. Nevertheless, to try to overcome this problem, we recruited only dogs older than 6 years. Another limitation was the use of a candidate gene approach, which permits comparison of allele frequencies of SNPs in genes suspected to be involved in SNA among dogs with the disease and others without (controls), but this method precludes finding other genes of interest. Genome-wide approaches would overcome this limitation, but the need for a large number of affected dogs and the high associated expense renders this difficult.

## Conclusions

The findings of the present study suggest that TLR2, 4 and 9 are not implicated in the pathogenesis of SNA in dogs. However, these results are limited by the size of the study population. This does not rule out the possibility of innate immune defects contributing to the development of the disease. Other *TLR* genes and/or other genes involved in mucosal immunity may be important and require further investigation.

## Methods

### Selection of single nucleotide polymorphisms

A mutational analysis of the *TLR2*, *4* and *9* exons was carried out in nine dogs diagnosed with SNA in order to determine the presence of non-synonymous SNPs in the coding regions of the genes and to investigate these further in a case-control association study to determine their significance in SNA.

The nine selected dogs (aged 10 months to 14 years; median 6.7 years; 3 females and 6 males) were from various breeds: Golden Retriever (n = 2), Labrador Retriever (n = 2), Rottweiler (n = 2), and one each of Beauceron, German Shepherd Dog and crossbreed. Diagnosis of SNA was made on the basis of physical examination, observation of intranasal fungal plaques associated with severe turbinate destruction on rhinoscopy, presence of serum antibody specific for *A. fumigatus* and culture of *A. fumigatus* from suspected lesions. A computed tomography examination of the head was performed in some dogs.

Blood stored in ethylenediaminetetraacetic acid (EDTA) or biopsy tissue from the nasal mucosa were available for each dog and were used for extraction of genomic DNA using the Macherey-Nagel NucleoSpin blood (or tissue) isolation kit (ABgene, Epsom, UK). All biological material used to perform the present study was collected for diagnostic purpose in all dogs. Nevertheless, the right to use it was obtained by owner consent.

Canine *TLR2*, *4* and *9* forward and reverse primers were designed via Primer 3 (http://bioinfo.ut.ee/primer3-0.4.0/primer3/), using the canine specific GenBank sequences, as described previously [27] (Table 7). Only primers for exon 3 were design for *TLR9*, because only two bases of exon 2 are coding. Primers were synthesized by Eurogentec S.A. (Seraing, Belgium) and reconstituted in TE buffer (10 mM Tris, 1 mM EDTA, pH 8) before use.

**Table 7:** **Primers used for amplifying** ***TLR2*,** ***4*** **and** ***9*** **for sequencing**

**Gene**	**Accession number**		**Product length**	**Forward primer (5′-3′)**	**Reverse primer (5′-3′)**
***TLR2***	*NC_006597.3*		2538	AATAAACTGTCAAAACAATCACTCA	AAGCAAGTCTGCAAAGGACA
***TLR4***	*NC_006593.3*	exon 1	359	ACAAAAGCCCAGAACGCTAA	TGCACAGAGAGCAGTTTTTCA
		exon 2	397	GAGAGAGGGCAGTTGAGGTG	AGATGAGGCAATGGGATCTG
		exon 3	3111	CTGAATCTGTGGGGCTTCTT	GAGACATGAAAAATGAGAACTGGA
***TLR9***	*NC_006602.3*	exon 3	3390	GGAACCCTGTTGGGAGACC	TTTGGGAAGGAAAGCCTGAC

Conventional polymerase chain reaction (PCR) was performed using Taq Platinum® PCR master mix (Invitrogen, California, USA), with reactions heated to 94°C for 2 min, followed by 35 cycles of 94°C for 25 sec, 60°C for 25 sec and 68°C for 4 min then a further 72°C for 7 min. The PCR products obtained were separated by 2% w/v agarose gel electrophoresis and appropriately sized products were excised with a clean scalpel blade and purified using the Nucleospin® Extract II (Macherey-Nagel, Hoerdt, France) as per the manufacturer’s instructions. Gel-purified PCR products were sent to GIGA research centre in Liège, Belgium for sequencing using sequencing primers specifically designed to cover the entire sequence of the exons (Table 8).

**Table 8:** Additional internal sequencing primers used

**Gene**	**Primer set**	**Primer sequence (5′-3′)**
***TLR2***	*TLR2* sequencing forward	TGGTTCCTTGCTCACTTTCA
	*TLR2* sequencing reverse	TGGCAAAATCAGGGAAAATG
***TLR4***	*TLR4* exon 3 sequencing forward	GGTGTCCCAGGAATCATTTG
	*TLR4* exon 3 sequencing reverse	TAGGATCTGGAGGGAGAGGAG
***TLR9***	*TLR9* exon 3 sequencing forward	GTTCAGCCGGAGATGTTTGT
	*TLR9* exon 3 sequencing reverse	CCAGCTTGTTATGGGACAGG

The sequence data were compared with the canine-specific GenBank sequences using GeneStudio contig assembly editor (http://www.genestudio.com/), and non-synonymous SNPs were identified.

### SNP genotyping

The non-synonymous SNPs identified in the first part of the study were further analysed in case and control populations by genotyping.

The case group consisted of 31 dogs diagnosed with SNA (aged 10 months to 12 years; median 6.5 years; 7 females and 24 males) from four different breeds: Golden Retriever (n = 10; minimum age 10 months; maximum age 12 years; median 7 years; 1 female and 9 males), Labrador Retriever (n = 8; minimum age 1.3 years; maximum age 12 years; median 7.1 years; 2 females and 6 males), Rottweiler (n = 9; minimum age 3 years; maximum age 9 years; median 5 years; 2 females and 7 males) and Beauceron (n = 4; minimum age 10 months; maximum age 10.5 years; median 2.5 years; 2 females and 2 males). Diagnosis of SNA was based on the criteria given above. These dogs were presented at the Veterinary Clinic of the University (CVU) of Liège (Belgium) between July 2007 and June 2013. During that period, a total of 72 dogs from various breeds were diagnosed with SNA at the same institution. The breeds of dogs included in the present study were the 4 most frequently encountered breeds in these 72 dogs. A Fisher’s exact test was used to assess whether those breeds were predisposed to the disease as compared with the general population of dogs presented to the CVU during the same period of time. The four canine breeds were proved to be predisposed to the disease (p < 0,01 in the Golden Retriever and the Rottweiler; p < 0,05 in the Labrador Retriever and the Beauceron).

The control group consisted of 31 dogs of the same breeds: Golden Retriever (n = 10; minimum age 6 years; maximum age 14 years; median 9 years; 4 females and 6 males), Labrador Retriever (n = 8; minimum age 9 years; maximum age 14 years; median 12 years; 3 females and 5 males), Rottweiler (n = 9; minimum age 6 years; maximum age 8 years; median 7.8 years; 4 females and 5 males) and Beauceron (n = 4; minimum age 8 years; maximum age 12 years; median 11 years; 1 female and 3 males), with no history or signs of respiratory disease. Inflammatory, immune-mediated, neoplastic or infectious diseases were also excluded. Twelve dogs were healthy; five were presented for laryngeal paralysis, four for degenerative osteoarthritis, three for a lipoma and two for perineal hernia. One dog each had vaginal haemorrhage, arrhythmia, anal sac impaction, hyperadrenocorticism or hypoadrenocorticism. All dogs in this group were older than 6 years of age (6 - 14 years; median 9 years; 15 females and 16 males). Owner consent was again obtained for each dog.

Blood stored in ethylenediaminetetraacetic acid (EDTA) was used for genomic DNA extraction using the Macherey-Nagel NucleoSpin Blood Isolation Kit (ABgene, Epsom, UK).

The allele-specific primer-based PCR was used to genotype the SNPs [28],[29]. Briefly, allele-specific PCR primers were designed so that the 3′ terminal nucleotide of a primer corresponded to the site of the SNPs (Table 9). An allele-specific primer matches perfectly with one allele (the specific allele) or has a 3′ mismatch with a non-specific allele. Because mismatched 3′ termini are extended by *Taq* polymerases with much lower efficiency than correctly matched termini, the allele-specific primer preferentially amplified the specific allele. To improve the specificity of traditional allele-specific primers, we followed a modified method [30],[31] in which an artificial base pair mismatch was introduced between the third and the fifth base from the 3′ terminus to each primer (Table 9). Two positive controls were designed for each SNP, to include allele-specific target sequence (without the addition mismatch) and the common primer (forward or reverse as appropriate), to test the ability of the assay to identify the SNP (Table 9). Primers and positive controls were synthesized by Eurogentec S. A. (Seraing, Belgium) and reconstituted in TE buffer (10 mM Tris, 1 mM EDTA, pH 8) before use.

**Table 9:** Allele-specific primers and positive control used for genotyping

**SNP**	**Primer**	**Primer sequence (5′-3′)**	**Positive control**
***TLR2***			
C137A	Forward	CCGCTCCAGATCTTTGAAgT**C**	CCGCTCCAGATCTTTGAACT**C**CATGCATTGGCAACAGTGACCTTCGGGATT
(110 bp) [60°C]		CCGCTCCAGATCTTTGAAgT**A**	CCGCTCCAGATCTTTGAACT**A**CATGCATTGGCAACAGTGACCTTCGGGATT
	Reverse	AATCCCGAAGGTCACTGTTG	
C1547T	Forward	GCCTCCTTCTTACCCACCTT	
(81 bp) [60°C]	Reverse	GGAATCCAGTTGCTCCTaC**G**	GGAATCCAGTTGCTCCTTC**G**AGAAAGAATTTGTAAGGTGGGTAAGAAGGAGGC
		GGAATCCAGTTGCTCCTaC**A**	GGAATCCAGTTGCTCCTTC**A**AGAAAGAATTTGTAAGGTGGGTAAGAAGGAGGC
***TLR4***			
C107T	Forward	GGAAAGGAGAGAGGGCAGTT	
(101 bp) [60°C]	Reverse	AGCTCCATGCATTGGTAAGaA**G**	AGCTCCATGCATTGGTAAGTA**G**GCATGCACCTCAACTGCCCTCTCTCCTTTCC
		AGCTCCATGCATTGGTAAGaA**A**	AGCTCCATGCATTGGTAAGTA**A**GCATGCACCTCAACTGCCCTCTCTCCTTTCC
T500C	Forward	CAATCTTATCCATTCCTTCAAcC**T**	CAATCTTATCCATTCCTTCAAGC**T**TGCGTATTTCTCTAACATGCCCAACCTGGAG
(58 bp) [60°C]		CAATCTTATCCATTCCTTCAAcC**C**	CAATCTTATCCATTCCTTCAAGC**C**TGCGTATTTCTCTAACATGCCCAACCTGGAG
	Reverse	CTCCAGGTTGGGCATGTTAG	
A600C	Forward	CTAACATGCCCAACCTGGAG	
(110 bp) [60°C]	Reverse	TAAAGAAAGGTTGAGTAGTGGCtT**T**	TAAAGAAAGGTTGAGTAGTGGCAT**T**TGATGTAGACGTTCTCCAGGTTGGGCATGTTAG
		TAAAGAAAGGTTGAGTAGTGGCtT**G**	TAAAGAAAGGTTGAGTAGTGGCAT**G**TGATGTAGACGTTCTCCAGGTTGGGCATGTTAG
A688G	Forward	GGTTCCTTTAAAGAAATTAAACTCgAT**A**	GGTTCCTTTAAAGAAATTAAACTCCAT**A**AACTGACTTTGAGAAGCCAGCCAGACCTTGAATA
(97 bp) [60°C]		GGTTCCTTTAAAGAAATTAAACTCgAT**G**	GGTTCCTTTAAAGAAATTAAACTCCAT**G**AACTGACTTTGAGAAGCCAGCCAGACCTTGAATA
	Reverse	AGCCAGCCAGACCTTGAATA	
G1039A	Forward	ATGGCAACGGTTGGAAATAG	
(66 bp) [60°C]	Reverse	AGAGAGTCCAGCTCCCAaG**C**	AGAGAGTCCAGCTCCCATG**C**CTAAGTTACAATTAACTATTTCCAACCGTTGCCAT
		AGAGAGTCCAGCTCCCAaG**T**	AGAGAGTCCAGCTCCCATG**T**CTAAGTTACAATTAACTATTTCCAACCGTTGCCAT
A1571T	Forward	ATGGCTGATAATTCCTTTCC	
(163 bp) [55°C]	Reverse	GGCTATTGTGACTCATATTTAaC**A**	GGCTATTGTGACTCATATTTATC**A**AGGGAGTTGTCCGGAAAGGAATTATCAGCCAT
		GGCTATTGTGACTCATATTTtTC**T**	GGCTATTGTGACTCATATTTATC**T**AGGGAGTTGTCCGGAAAGGAATTATCAGCCAT
G1807A	Forward	TGGGTCAAAGACCACAGACA	
(53 bp) [60°C]	Reverse	GCACACACCATTTGTTCAAgTT**C**	GCACACACCATTTGTTCAACTT**C**CACCAAGAGCTGTCTGTGGTCTTTGACCCA
		GCACACACCATTTGTTCAAgTT**T**	GCACACACCATTTGTTCAACTT**T**CACCAAGAGCTGTCTGTGGTCTTTGACCCA
***TLR9***			
G1138A	Forward	GCGCAGACTCAACCTGTCCTT	
(152 bp) [55°C]	Reverse	GCGACTGGAGCGTGGTgT**C**	GCGACTGGAGCGTGGTCT**C**CTTATGATAATTGAAGGACAGGTTGAGTCTGCGC
		GCGACTGGAGCGTGGTgT**T**	GCGACTGGAGCGTGGTCT**T**CTTATGATAATTGAAGGACAGGTTGAGTCTGCGC
A1372C	Forward	GCGGCTGCCACAGGGGAGGT	
(95 bp) [60°C]	Reverse	GAGCCGGGGGTGCCCAGaG**T**	GAGCCGGGGGTGCCCAGTG**T**CACAGTCTGCCTCTACCTCCCCTGTGGCAGCCGC
		AGCCGGGGGTGCCCAGaG**G**	GAGCCGGGGGTGCCCAGTG**G**CACAGTCTGCCTCTACCTCCCCTGTGGCAGCCGC
A2158G	Forward	CTGGACCTCAGCGGCAACAG	
(58 bp) [60°C]	Reverse	CGGCCAGGGCAAAAAAcC**T**	CGGCCAGGGCAAAAAAGC**T**CACGAAGCCGATGCTGTTGCCGCTGAGGTCCAG
		CGGCCAGGGCAAAAAAcC**C**	CGGCCAGGGCAAAAAAGC**C**CACGAAGCCGATGCTGTTGCCGCTGAGGTCCAG
G2927A	Forward	CTCCTGCGTGCCAGCTTCCT	
(113 bp) [55°C]	Reverse	AGCCGCACATAGCGGGtG**C**	AGCCGCACATAGCGGGAG**C**CTGTTGGGCCAGCAGGAAGCTGGCACGCAGGAG
		AGCCGCACATAGCGGGtG**T**	AGCCGCACATAGCGGGAG**T**CTGTTGGGCCAGCAGGAAGCTGGCACGCAGGAG

The polymorphic base is in bold, the additional inserted 3′ mismatch is in lowercase, the amplicon size in base pairs (bp) and annealing temperature [°C] used for the qPCT are shown below the SNP name.

**Figure 1:**
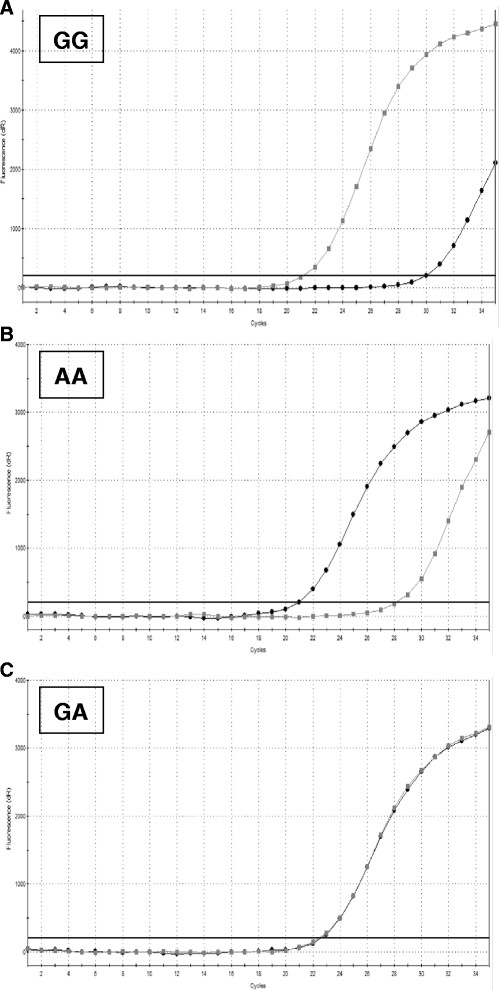
**Real time PCR results for** ***TLR4 A688G*** **SNP genotyping.** Figures 1**A** and **B** show the results for homozygous dogs; a lower Ct is obtained with the specific primer set and a higher Ct with the non-specific primer set. Figure 1**C** shows the results for a heterozygous dog; both assays give approximately equal Ct results.

The allele-specific reactions were performed by quantitative PCR (qPCR) using SYBR Green I with separate reactions for each primer set (two sets per SNP). SNP genotyping was performed by comparison of Ct values (Figure 1) with a homozygous sample having a lower Ct with the specific primer set and a higher Ct with the non-specific one, and a heterozygous sample having approximately equal results from both assays. Each assay was optimized with the synthetic positive controls and residual DNA from the samples used in the sequencing study. The qPCR was performed using GoTaq Colourless Master Mix (Promega Corporation, Southampton, UK) in a Stratagene Mx3005P (Agilent Technologies, Stockport, Cheshire, UK) with results analysed using the MxPro Software (ver. 4.1; Agilent Technologies). Optimisation involved varying the annealing temperature (55, 60 and 65°C), with and without additional MgCl_2_ (final concentration: 3.0 and 4.5 mM), to balance the Ct value difference between the target and non-target allele-specific PCR, and non-specific product formation with genomic DNA and water negative controls. The final reactions consisted of 200nM of each primer, 4.5 mM Mg and 1:50,000 SYBR Green I (Invitrogen) with a thermocycling protocol of incubation at 95°C for 2 min for Taq activation and then 35 cycles of 95°C for 15 sec and annealing (55 or 60°C: Table 9) for 30 sec during which the fluorescence data were collected. The thermocycling protocol was extended by heating samples from 75°C to 95°C in 0.5°C increments for 15 sec, during which time the fluorescence data were collected in order to create a melt curve. For each SNP, the two genotyping assays were run for a single DNA sample on the same plate which also included a number of additional controls including the target and non-target synthetic positive control, and AE buffer (negative control).

**Figure 2:**
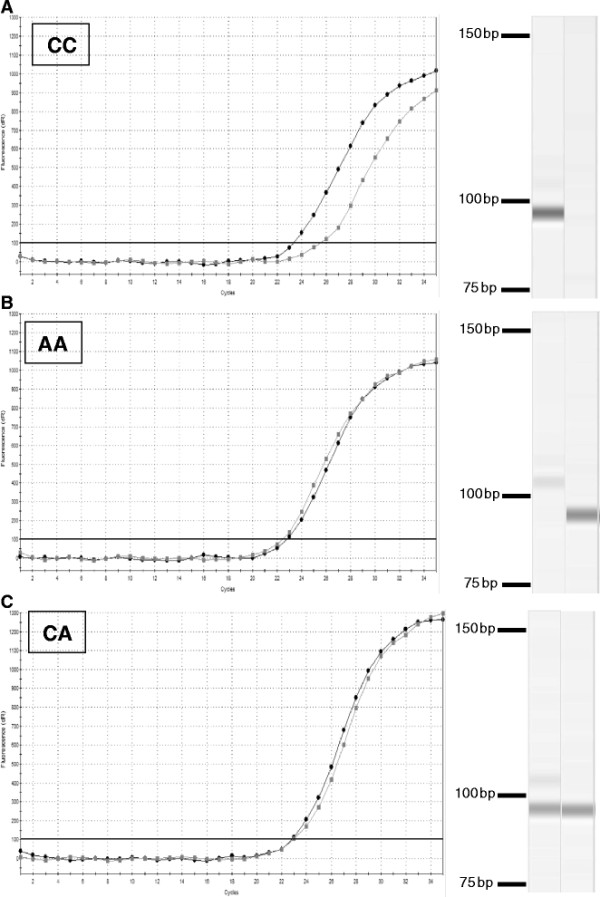
**Real time PCR and capillary electrophoresis results for** ***TLR9 A1372C*** **SNP genotyping.** For this SNP, genotyping was not possible on the basis of Ct value alone because Ct values were too similar with both homozygous (Figure 2**A** and **B**) and heterozygous (Figure 2**C**) dogs. Capillary electrophoresis of PCR products permits differentiation of the products according to their size.

For three SNPs (*TLR9 A1372C*, *TLR9 C2607T*, *TLR9 G2927A*) it was not possible to genotype on the basis of Ct value alone because Ct values were too similar with both homozygous and heterozygous animals due to the presence of additional amplified products in both reactions (Figure 2). Therefore, capillary electrophoresis (QIAxcel Advanced System, QIAGEN Group, UK) using the QIAxcel DNA High Resolution Kit (QIAGEN), QX Alignment Marker 15 bp/500 bp (QIAGEN) and QX DNA Size Marker 25–500 bp (QIAGEN) was used to differentiate the products according to their size (Figure 2) and to genotype each sample. Samples were analysed using the QIAxcel ScreenGel Software (ver. 1.2.0; QIAGEN) and the standard DM500 analysis protocol.

For one TLR4 SNP (*T23C*) we could not successfully optimise a set of allele-specific primers which could successfully genotype this base position. Both primers with and without the additional artificial base pair mismatch were tried, but an assay which would amplify DNA from dogs with the C allele could not optimised. Therefore, a Taq-man (hydrolysis) probe-based qPCR assay was designed using Primer3 and optimised against the synthetic positive control and residual DNA from the sequencing experiments. The assay comprised a forward primer (GCACAGAAAATGCCAGAATG), reverse primer (AGGTGAACCGACATGAAACC) and two allele-specific probes (FAM-CTACCCGCCTGG**C**TGGGATAT-BHQ-1) and (HEX-CTACCCGCCTGG**T**TGGGATAT-BHQ-1). The genotyping was performed using GoTaq Colourless Master Mix (Promega) with 200nM primers, 100nM of each probe and 4.5 mM Mg in a final volume of 25 μl. The same thermocycling protocol was used as for the allele-specific genotyping with a 60°C annealing temperature, but 40 cycles were performed with genotyping based on end-point fluorescence using the MxPro software. Reactions were run in duplicate for each sample with homozygous, heterozygous and no-template controls were run with each reaction.

### Association study

Statistical analysis was performed with the whole genome association analysis toolset PLINK (http://pngu.mgh.harvard.edu/~purcell/plink/). Minor allele frequencies were calculated for each SNP and case-control comparisons were analysed for all breeds together (using contingency tables and Chi-squared tests) and separately (using two-tailed Fisher exact tests). Statistical significance was set at p <0.05.

## Abbreviations

SNA: Sino-nasal aspergillosis

SNP(s): Single nucleotide polymorphism(s)

TLR(s): Toll-like receptor(s)

PAMPs: Pathogen-associated molecular patterns

CRS: Chronic rhinosinusitis

IA: Invasive aspergillosis

PCR: Polymerase chain reaction

qPCR: quantitative polymerase chain reaction

CTL: Control

## Competing interests

The authors declare that they have no competing interests.

## Authors’ contributions

EM contributed to study design, performed the experiments, interpreted the results and wrote the manuscript. IRP contributed to study design and the experiments, helped with laboratory work and interpretation of the results and revised the final manuscript. FF helped with study design and performed the statistical analysis. RL and CC participated in the sample collection. MJD revised the final manuscript. DP supervised study design and revised the final manuscript. All the authors read and approved the final manuscript.

## References

[B1] Ooi EH Wormald PJ Tan LW Innate immunity in the paranasal sinuses: a review of nasal host defenses Am J Rhinol 2008 22 1 13 19 10.2500/ajr.2008.22.3127 18284853

[B2] Vroling AB Fokkens WJ van Drunen CM How epithelial cells detect danger: aiding the immune response Allergy 2008 63 9 1110 1123 10.1111/j.1398-9995.2008.01785.x 18699929

[B3] Schroder NW Schumann RR Single nucleotide polymorphisms of Toll-like receptors and susceptibility to infectious disease Lancet Infect Dis 2005 5 3 156 164 10.1016/S1473-3099(05)01308-3 15766650

[B4] Braedel S Radsak M Einsele H Latgé J-P Michan A Loeffler J Haddad Z Grigoleit U Schild H Hebart H Aspergillus fumigatus antigens activate innate immune cells via toll-like receptors 2 and 4 Br J Haematol 2004 125 3 392 399 10.1111/j.1365-2141.2004.04922.x 15086422

[B5] Mambula SS Sau K Henneke P Golenbock DT Levitz SM Toll-like receptor (TLR) signaling in response to Aspergillus fumigatus J Biol Chem 2002 277 42 39320 39326 10.1074/jbc.M201683200 12171914

[B6] Meier A Kirschning CJ Nikolaus T Wagner H Heesemann J Ebel F Toll-like receptor (TLR) 2 and TLR4 are essential for Aspergillus-induced activation of murine macrophages Cell Microbiol 2003 5 8 561 570 10.1046/j.1462-5822.2003.00301.x 12864815

[B7] Romani L Immunity to fungal infections Nat Rev Immunol 2004 4 1 1 23 14661066 10.1038/nri1255

[B8] Ramaprakash H Ito T Standiford TJ Kunkel SL Hogaboam CM Toll-like receptor 9 modulates immune responses to Aspergillus fumigatus conidia in immunodeficient and allergic mice Infect Immun 2009 77 1 108 119 10.1128/IAI.00998-08 18936185 PMC2612288

[B9] Ramirez-Ortiz ZG Specht CA Wang JP Lee CK Bartholomeu DC Gazzinelli RT Levitz SM Toll-like receptor 9-dependent immune activation by unmethylated CpG motifs in Aspergillus fumigatus DNA Infect Immun 2008 76 5 2123 2129 10.1128/IAI.00047-08 18332208 PMC2346696

[B10] Carvalho A Pasqualotto AC Pitzurra L Romani L Denning DW Rodrigues F Polymorphisms in toll-like receptor genes and susceptibility to pulmonary aspergillosis J Infect Dis 2008 197 4 618 621 10.1086/526500 18275280

[B11] Sharp NJH Harvey CE O’Brien JA Treatment of canine nasal aspergillosis/penicilliosis with fluconazole (UK-49,858) J Small Anim Pract 1991 32 10 513 516 10.1111/j.1748-5827.1991.tb00868.x

[B12] Peeters D Clercx C Update on canine sinonasal aspergillosis Vet Clin North Am Small Anim Pract 2007 37 5 901 916 vi 10.1016/j.cvsm.2007.05.005 17693205

[B13] Peeters D Peters IR Clercx C Day MJ Quantification of mRNA encoding cytokines and chemokines in nasal biopsies from dogs with sino-nasal aspergillosis Vet Microbiol 2006 114 3–4 318 326 10.1016/j.vetmic.2005.11.065 16387453

[B14] Day MJ Canine sino-nasal aspergillosis: parallels with human disease Med Mycol 2009 47 s1 S315 S323 10.1080/13693780802056038 18608893

[B15] Park CS Cho JH Park YJ Toll-like receptor 2 gene polymorphisms in a Korean population: association with chronic rhinosinusitis Otolaryngol Head Neck Surg 2011 144 1 96 100 10.1177/0194599810390881 21493395

[B16] Bochud PY Chien JW Marr KA Leisenring WM Upton A Janer M Rodrigues SD Li S Hansen JA Zhao LP Aderem A Boeckh M Toll-like receptor 4 polymorphisms and aspergillosis in stem-cell transplantation N Engl J Med 2008 359 17 1766 1777 10.1056/NEJMoa0802629 18946062 PMC2656610

[B17] de Boer MG Jolink H Halkes CJ van der Heiden PL Kremer D Falkenburg JH van de Vosse E van Dissel JT Influence of polymorphisms in innate immunity genes on susceptibility to invasive aspergillosis after stem cell transplantation PLoS ONE 2011 6 4 e18403 10.1371/journal.pone.0018403 21483748 PMC3070725

[B18] Mercier E Peters IR Day MJ Clercx C Peeters D Toll- and NOD-like receptor mRNA expression in canine sino-nasal aspergillosis and idiopathic lymphoplasmacytic rhinitis Vet Immunol Immunopathol 2012 145 3-4 618 624 10.1016/j.vetimm.2012.01.009 22321737

[B19] Jungi TW Farhat K Burgener IA Werling D Toll-like receptors in domestic animals Cell Tissue Res 2011 343 1 107 120 10.1007/s00441-010-1047-8 20927536

[B20] Kathrani A House A Catchpole B Murphy A German A Werling D Allenspach K Polymorphisms in the TLR4 and TLR5 gene are significantly associated with inflammatory bowel disease in German shepherd dogs PLoS ONE 2010 5 12 e15740 10.1371/journal.pone.0015740 21203467 PMC3009732

[B21] Irion DN Schaffer AL Famula TR Eggleston ML Hughes SS Pedersen NC Analysis of genetic variation in 28 dog breed populations with 100 microsatellite markers J Hered 2003 94 1 81 87 10.1093/jhered/esg004 12692167

[B22] Carvalho A Cunha C Carotti A Aloisi T Guarrera O Di Ianni M Falzetti F Bistoni F Aversa F Pitzurra L Rodrigues F Romani L Polymorphisms in Toll-like receptor genes and susceptibility to infections in allogeneic stem cell transplantation Exp Hematol 2009 37 9 1022 1029 10.1016/j.exphem.2009.06.004 19539691

[B23] Kesh S Mensah NY Peterlongo P Jaffe D Hsu K VDB M O’Reilly R Pamer E Satagopan J Papanicolaou GA TLR1 and TLR6 polymorphisms are associated with susceptibility to invasive aspergillosis after allogeneic stem cell transplantation Ann N Y Acad Sci 2005 1062 95 103 10.1196/annals.1358.012 16461792

[B24] Tewfik MA Bosse Y Hudson TJ Vallee-Smejda S Al-Shemari H Desrosiers M Assessment of Toll-like receptor 2 gene polymorphisms in severe chronic rhinosinusitis J Otolaryngol Head Neck Surg 2008 37 4 552 558 19128592

[B25] Vercelli D Genetics, epigenetics, and the environment: switching, buffering, releasing J Allergy Clin Immunol 2004 113 3 381 386 quiz 387 10.1016/j.jaci.2004.01.752 15007332

[B26] Terwilliger JD Goring HH Gene mapping in the 20th and 21st centuries: statistical methods, data analysis, and experimental design 2000 Hum Biol 2009 81 5-6 663 728 10.3378/027.081.0615 20504191

[B27] Peters IR Helps CR Hall EJ Day MJ Real-time RT-PCR: considerations for efficient and sensitive assay design J Immunol Methods 2004 286 1–2 203 217 10.1016/j.jim.2004.01.003 15087233

[B28] Kwok S Kellogg DE McKinney N Spasic D Goda L Levenson C Sninsky JJ Effects of primer-template mismatches on the polymerase chain reaction: human immunodeficiency virus type 1 model studies Nucleic Acids Res 1990 18 4 999 1005 10.1093/nar/18.4.999 2179874 PMC330356

[B29] Drenkard E Richter BG Rozen S Stutius LM Angell NA Mindrinos M Cho RJ Oefner PJ Davis RW Ausubel FM A simple procedure for the analysis of single nucleotide polymorphisms facilitates map-based cloning in Arabidopsis Plant Physiol 2000 124 4 1483 1492 10.1104/pp.124.4.1483 11115864 PMC1539302

[B30] Hayashi K Hashimoto N Daigen M Ashikawa I Development of PCR-based SNP markers for rice blast resistance genes at the Piz locus Theor Appl Genet 2004 108 7 1212 1220 10.1007/s00122-003-1553-0 14740086

[B31] Papp AC Pinsonneault JK Cooke G Sadee W Single nucleotide polymorphism genotyping using allele-specific PCR and fluorescence melting curves Biotechniques 2003 34 5 1068 1072 12765033 10.2144/03345dd03

